# Synovial chondromatosis of the hip with atypical MRI morphology and mistakable clinical symptoms — a case report

**DOI:** 10.3109/17453674.2011.570679

**Published:** 2011-04-05

**Authors:** Jan C Schagemann, Peter Hunold, Martin Russlies, Hagen Mittelstaedt

**Affiliations:** ^1^Clinic for Musculoskeletal Surgery, Section for Orthopedics; ^2^Clinic for Radiology and Nuclear Medicine, University Medical Center Schleswig-Holstein, Campus Lübeck, Lübeck, Germany

A 32-year-old male presented to us with 6-month symptoms of unilateral spinal claudication (L3 right) together with lumbar pain and intermittent tenderness of the right sacroiliac joint with insidious onset and slow progression. The pain was also occasionally localized to the anterior groin or the lateral aspect of the right hip including sporadic indolent popping. Pain relief was exclusively achieved on spine flexion or positioning of the hip in a flexed and externally rotated position. The patient reported episodes of exacerbation, which could be followed by completely asymptomatic periods in which he was capable of participating in all activities, and bicycling in particular. Later, there was diffuse right thigh discomfort at night.

Physical examination revealed a slight, uncomfortable limitation of range of motion of the right hip. A lumbar MRI was performed in order to exclude a spinal stenosis or nerve root compression. This was normal. Radiographs of the hip showed regular neck-shaft and center-edge angles, and a mild Pinzer-type of femoroacetabular impingement without major degenerative changes. No bone erosion, joint space alterations, or radiopaque bodies were apparent.

A subsequent MRI of the hips showed the right joint space filled with a homogenous tissue that appeared slightly hyperintense in pre-contrast T1-weighted TSE images compared to muscle (Figure 1). The mass was hyperintense and slightly inhomogenous in fat-suppressed T2-weighted TSE images (STIR). It appeared to originate from the inferior joint capsule expanding into the joint space. No signal loss corresponding to calcified areas, joint effusion, or a ruptured ligamentum capitis femoris could be found. The acetabular fossa was slightly eroded. In the gadolinium-enhanced T1-weighted TSE images with fat suppression (Figure 2), the tissue and the thickened synovia showed slight enhancement with a stronger enhancing rim surrounding the mass. There was no enhancement or edema of the joint surrounding soft tissue or bone.

**Figure 1.**
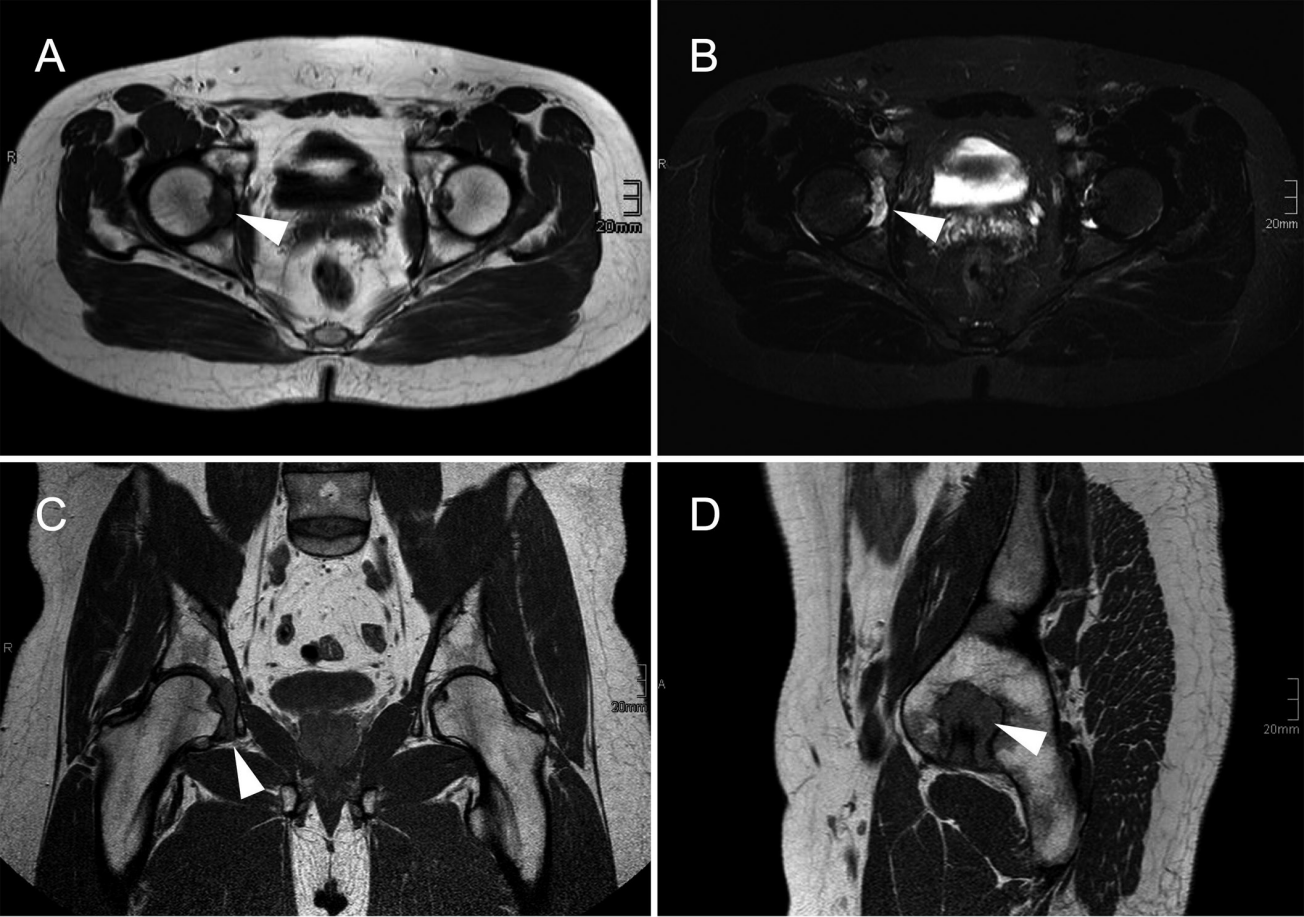
Un-enhanced MR images acquired with a T1-weighted TSE sequence in transversal (A), coronal (C), and sagittal (D) orientation. The tissue within the joint space (arrows) appeared hyperintense, comparable to skeletal muscle, and seemed to arise from the caudal parts of the joint's capsule. The ligamentum capitis femoris was well differentiated from the mass. In the fat-suppressed T2-weighted TSE images (B), inhomogenous hyperintensity of the mass was apparent. Remarkably, there was no signal loss within the mass indicating areas of calcification.

**Figure 2.**
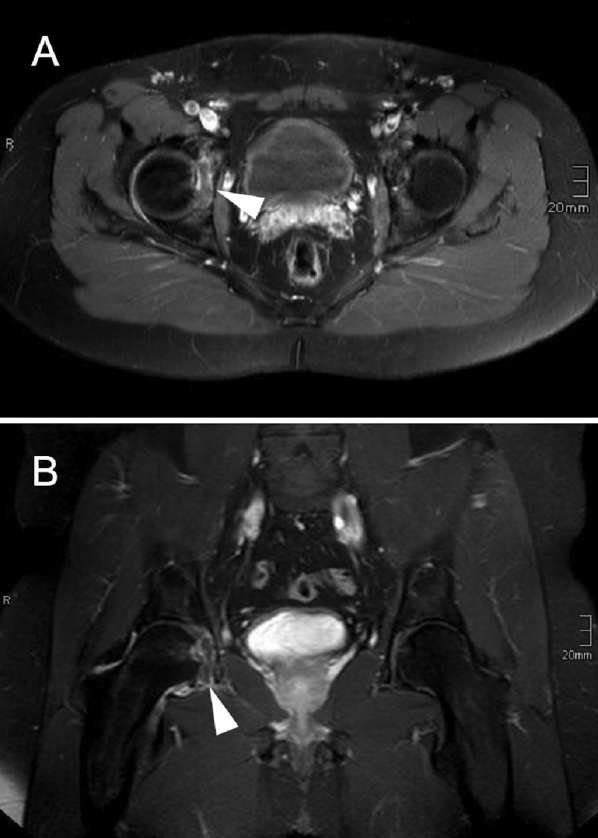
Gadolinium-enhanced MR images acquired with a fat-suppressed T1-weighted TSE sequence in transversal (A) and coronal orientation (B). Arrows show the minimally enhancing mass with a strong rim enhancement and a suggested relation to the caudal synovia. Notice the slight enhancement of the joint's capsule without the presence of effusion.

An un-enhanced thin-slice CT scan with coronal reformats revealed a slightly widened joint space of the right femoroacetabular joint that was filled with a homogenous soft tissue-isodense material instead of fat and synovial fluid, as seen on the contralateral side (Figure 3). No calcifications could be found within the soft tissue. Slight erosion of the acetabular fossa was evident.

**Figure 3.**
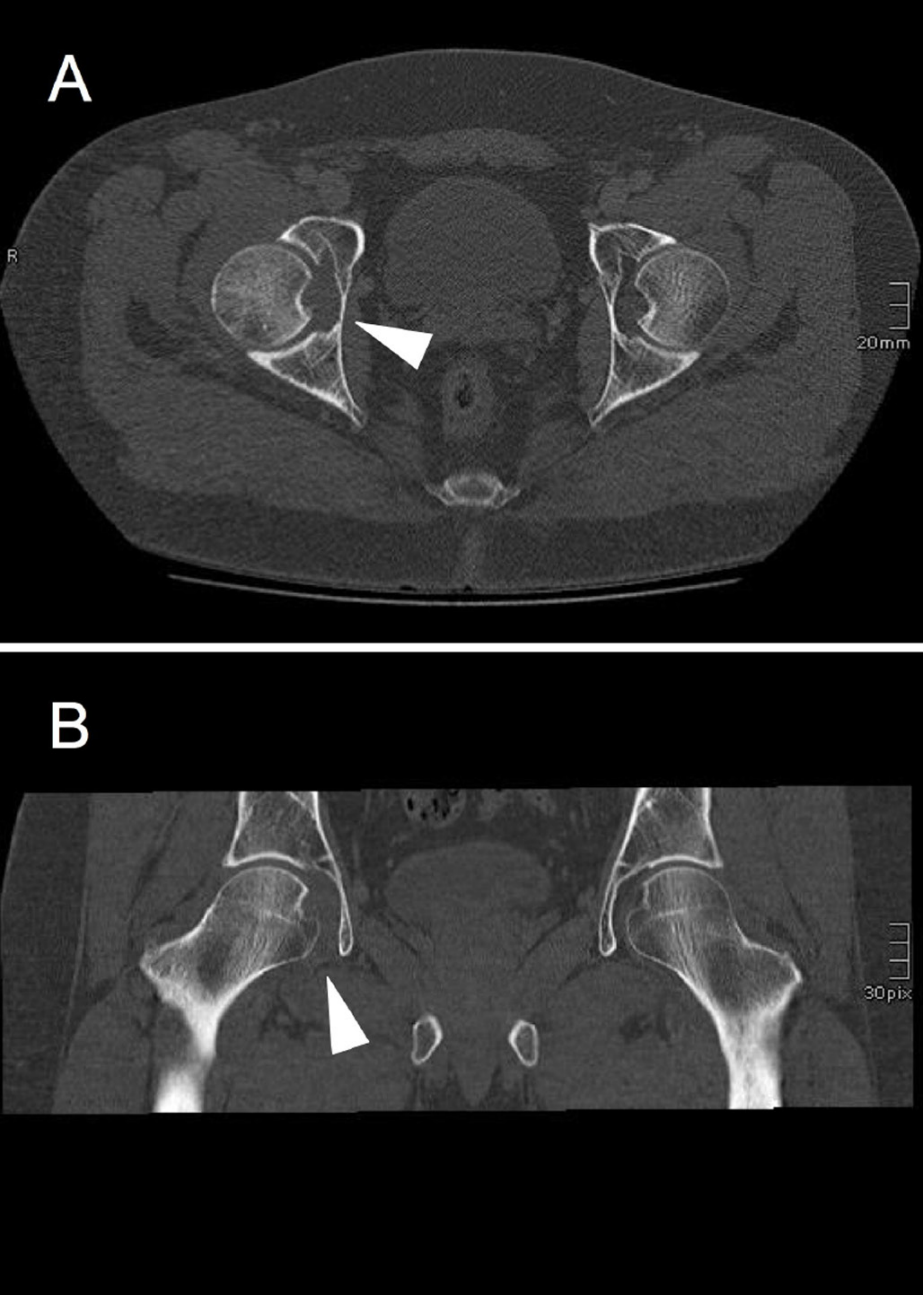
Un-enhanced thin-slice CT of the hips in transverse-oriented source images (A) and coronal reformats (B). The arrows show the soft tissue-isodense mass, which filled up the right joint space and led to a widening of the joint space compared to the contralateral side. No calcifications or massive extrinsic bone erosions could be seen.

Since all diagnostic modalities were inconclusive, an open biopsy using a minimally invasive anterolateral approach to the hip was done and revealed numerous intra-articular cartilaginous bodies. Histology verified the diagnosis of a synovial chondromatosis without any signs of malignancy. By a later arthroscopy, loose bodies were removed including a synovectomy. The patient recovered well from surgery and was discharged for outpatient rehabilitation including CPM. At 1-year follow-up, the patient was free from symptoms and had no physical signs of local recurrence.

## Discussion

Synovial chondromatosis is usually a monoarticular process that most commonly affects large joints—primarily the knee and less frequently the hip—of men in their forties (Maurice et al. 1988, Shpitzer et al. 1990, Lim et al. 2006, Fuerst et al. 2009). It is characterized by cartilaginous nodule formation secondary to synovial metaplasia. These nodules may undergo calcification (osteochondromatosis) and detach from the synovia. Malignant transformation is uncommon, and its etiology is not completely understood (Bertoni et al. 1991, Davis et al. 1998, Fuerst et al. 2009). Clinical symptoms are often unspecific, which makes synovial chondromatosis a difficult diagnosis in its early phases. It may progress over several years until the osteochondral fragments are detected on radiological imaging as radiopaque intra-articular bodies (Shpitzer et al. 1990, Knoeller 2001, Chen et al. 2003) that may be associated with bony erosions (Goldberg et al. 1983, Knoeller 2001, Kim et al. 2002, Lim et al. 2006, Murphey et al. 2007).

Synovial chondromatosis of the hip usually leads to symptoms that are projected to the anterior groin and/or lateral aspect of the hip. Pain, swelling, and restricted range of motion are supposed to be the primary symptoms; crepitus, popping, or even catching caused by intra-articular bodies are not uncommon (Trias and Quintana 1976, Roulot and Le Viet 1999, Butt et al. 2005). Typically, positioning of the hip in a flexed and externally rotated position relieves the pain because of the reduced strain derived from the capsule. In our case, however, lumbar pain and most of all spinal claudication-like (and thus misleading) symptoms prevailed.

According to Murphey et al. (2007), radiographs show pathognomonic radiopaque bodies in most cases (70–95%) of primary chondromatosis. This was not the case in our patient. In addition, typical radiological signs such as joint space alterations and bone erosions were also absent. The MRI revealed a homogenous joint space-filling tissue with a slight hyperintense signal in T1-weighted TSE images and in fat-suppressed T2-weighted TSE images. The tissue, but also the thickened synovia, was enhanced in the post-contrast T1-weighted TSE images with fat suppression, with a stronger enhancing rim. Peripheral but not septal or lobulated enhancement was found in post-contrast images that would have indicated avascular and therefore non-enhancing cartilaginous nodules (Kramer et al. 1993, Frick et al. 2007). According to Kramer et al. (1993), synovial chondromatosis without characteristic focal areas of low signal intensity corresponding to loose bodies (3/21 cases) or with high-signal-intensity foci that are isointense relative to fat with a peripheral low-intensity rim (2/21 cases) is uncommon.

These findings have recently been supported by Murphey et al. (2007), including several variations in signal pattern. For example, the signal intensity on T1-weighted images of non-calcified areas is often lower than that of muscle and similar to the signal intensity of fluid (and not intermediate). The authors assumed that long TR images might occasionally contain small foci of slightly lower signal intensity within areas of overall high signal intensity, revealing small chondroid bodies that have not yet been calcified. However, the signal pattern characteristic of and predominant in synovial chondromatosis i.e. low to intermediate intensity with T1-weighting and very high intensity with T2-weighting, with focal areas of low intensity corresponding to hypointense calcifications (Kramer et al. 1993, Frick et al. 2007), was lacking in our case. Besides, the tissue seemed to originate from the inferior joint capsule expanding into the joint space that resembled an intra-articular ganglion. Extra-articular extension was not seen, although it is often associated with synovial chondromatosis of the hip (Kim et al. 2002, Robinson et al. 2004).

The subsequent CT scan showed slight erosion of the acetabular fossa and a widened joint space compared to the contralateral side. It is generally agreed that MRI and CT are the best imaging modalities for diagnosis of a synovial chondromatosis (Goldberg et al. 1983, Knoeller 2001, Kim et al. 2002, Lim et al. 2006, Murphey et al. 2007), yet the sensitivity is still strongly dependent on the histological nature of the lesion—and most of all the degree of calcification. The vast majority of synovial chondromatosis cases show the latter (Murphey et al. 2007). In our case, exclusively moderate synovial thickening and slight bone erosions suggested this diagnosis. These alterations are quite characteristic, yet not conclusive (Kim et al. 2002). Moreover, one might have expected more progressive extrinsic erosion of bone, which is supposed to be pathognomonic for less capacious joints such as the hip (Goldberg et al. 1983, Norman and Steiner 1986, Murphey et al. 2007).

It is generally agreed that a complete synovectomy is required for successful treatment and prevention of recurrence. Synovectomy can be done arthroscopically or by open arthrotomy with or without dislocation. Although open procedures provide the lowest recurrence rate, complications such as osteonecrosis of the femoral head may occur (Lim et al. 2006). Thus, a decision must be made on whether to accept complications in order to achieve complete synovectomy or to risk recurrence after an arthroscopic procedure. This issue remains controversial (Boyer and Dorfmann 2008, Nakamura et al. 2009).
